# A Growing Problem: Increases in Child‐Appealing Marketing on Infant and Young Children's Foods in Australia (2015 vs 2024)

**DOI:** 10.1111/mcn.70226

**Published:** 2026-07-14

**Authors:** Prisca Petty Arfines, Daisy Coyle, Eden Barrett, Jennifer McCann, Elizabeth Dunford, Alexandra Jones

**Affiliations:** ^1^ Food Policy Division, The George Institute for Global Health University of New South Wales Sydney New South Wales Australia; ^2^ Institute for Physical Activity and Nutrition, School of Exercise and Nutrition Sciences Deakin University Geelong Victoria Australia; ^3^ Department of Nutrition, Gillings Global School of Public Health The University of North Carolina at Chapel Hill Chapel Hill North Carolina USA

**Keywords:** food analysis, food labelling, food packaging, infant food, marketing, nutrition labelling, processed food

## Abstract

Commercial foods for infants and young children are widely available and often introduced early, including before 6 months, raising concerns about their often high levels of sugar and sodium, limited micronutrient content, and sweet flavours that may shape future taste preferences. Product packaging further influences caregiver choices through child‐appealing images and claims. This study examined changes in child‐appealing marketing appearing on packaged commercial infant and young children's foods available in Australia in 2015 (*n* = 311) and 2024 (*n* = 298). Products were coded using a validated framework comprising 11 core techniques (child‐targeted, e.g., bright colours, cartoon characters, fun themes) and 9 broad techniques (caregiver‐targeted, e.g., nutrition/health, texture claims). Marketing power was calculated as the sum of techniques per product. The proportion of products featuring child‐appealing marketing increased from 73.0% in 2015 to 89.9% in 2024, with significant increases in appeals to fun (48.2% to 79.2%), branded characters (33.8% to 62.4%), and child‐appealing visuals on the package (60.5% to 72.5%). Claims regarding texture were high in both years (99.7% in 2015 and 100.0% in 2024), and there was a notable growth in messages promoting value, convenience, or sustainability (from 50.2% to 62.1%). Marketing power scores rose significantly over time, indicating an intensification of persuasive techniques on packaging. These findings highlight systemic commercial strategies that promote nutritionally poor foods to infants, young children and caregivers, undermining public health efforts. Stronger, government‐led regulation is urgently needed to address the widespread use of both child‐targeted and caregiver‐targeted marketing on commercial foods for infants and young children.

## Summary

- Child‐appealing marketing on baby food packaging rose from 73% in 2015 to 89.9% in 2024.
- A marked rise in core child‐targeted techniques was observed, including branded characters, playful visuals, themes, and graphics.
- Broad caregiver‐targeted techniques, such as texture claims, have remained high since 2015, while value, convenience, philanthropy, and sustainability claims increased.
- Intensification of child‐appealing marketing across categories, packaging types, and ultra‐processed foods highlights food system issues undermining public health.
- Findings show gaps in Australia's regulatory framework and highlight stronger government‐led regulation of child‐ and caregiver‐targeted marketing for foods for infants and young children.

## 1 Introduction

The World Health Organization (WHO) and Australia's National Health and Medical Research Council (NHMRC) recommend exclusive breastfeeding for the first 6 months of a child's life, followed by continued breastfeeding with solid foods for at least 12 months and up to 2 years or longer, depending on the mother and child's preference (World Health Organization 2023a; National Health and Medical Research Council NHMRC 2013). Despite these recommendations, the consumption of commercially produced foods for infants and young children continues to rise. Global sales of baby foods (excluding infant formula) increased from US$9.6 billion in 2010 to US$17.9 billion in 2022 (Dunford and Popkin 2023). Research shows that commercial foods for infants are introduced as early as 4 months of age (Fox et al. 2004; Grummer‐Strawn et al. 2008), and most categories, including cereals, snacks, and ready‐to‐eat meals, are dominated by ultra‐processed foods (UPFs) (Santos et al. 2022). In many developed countries, purchases of commercial foods for older infants (aged 6–12 months) and young children (aged 12–36 months) are high, as parents and caregivers rely on these products for their convenience, shorter preparation time, and the perception that they are healthy options for their children (Maslin and Venter 2017; Hollinrake et al. 2024). However, growing concerns have been raised about the appropriateness and nutritional quality of these products, including limited micronutrient bioavailability, high sugar and sodium content, smooth texture, and predominantly sweet flavour profile (Maslin and Venter 2017; Moumin et al. 2020; Antignani et al. 2022). In addition, the narrow range of vegetables used limits dietary diversity (Bernal et al. 2021).

The food industry employs persuasive marketing techniques to influence consumer behaviour and purchasing decisions (Jenkin et al. 2014). These include a wide range of advertising avenues, spanning traditional media like television to increasingly modern digital platforms such as the internet, social media, and mobile apps. Other strategies involve point‐of‐sale tactics, such as discounts, school‐based promotions (including branded events or sponsored materials), sponsorships, and product placement (Hawkes 2004). Among these forms of marketing, product packaging has emerged as a particularly effective channel for capturing consumer attention based on visual cues, especially among children (Hallez et al. 2020; Smith et al. 2019). Product packaging often features visual elements that appeal to children, such as cartoon characters, bright colours, and playful language, alongside on‐pack claims aimed at persuading parents (Hallez et al. 2020; Leonard et al. 2019).

In the Australian context, previous studies have indicated that products for infants and young children (up to 36 months) are among the most heavily targeted categories for on‐pack marketing (Jones et al. 2023), with up to 87% of products displaying child‐appealing marketing (Chung et al. 2023). Child‑appealing marketing is an umbrella term that comprises two sets of techniques: core techniques that target children directly through elements such as cartoon characters and colourful or playful imagery; and broad techniques that target caregivers through on‑pack claims, including nutrition or health claims and cues related to texture or developmental suitability (Mulligan et al. 2021). A 2024 study reported that 100% of Australian commercial infant and young children's products failed to comply with recommendations outlined in the WHO Nutrient and Promotion Profile Model (NPPM) (Dunford et al. 2024). The NPPM is an international framework that operationalises principles articulated in the WHO Guidance on ending inappropriate promotion of foods for infants and young children by providing nutrient‑profiling criteria and standards for appropriate promotional practices. These recommendations cover a wide range of promotional practices, including restrictions on child‑appealing marketing, with Australian results highlighting a continued gap between current practice and internationally endorsed public health guidance (World Health Organization Regional Office for Europe 2022). Packaging strategies that combine core techniques targeting children and broad techniques targeting caregivers are particularly influential in retail environments, where purchasing decisions are often made impulsively (Silayoi and Speece 2004). These practices highlight the need for closer scrutiny of product packaging as a marketing tool and its role in shaping perceptions and choices around foods for infants and young children.

While many countries have introduced policies to limit unhealthy food marketing to children, most remain voluntary and limited in scope (World Health Organization 2022). As of May 2022, around 60 countries have adopted restrictions, with only 20 implementing mandatory policies, while others focus on school settings. Policies vary, covering all foods or specific items like sugary drinks, but few focus on marketing to infants and young children. Regulations specifically targeting unhealthy food marketing for infants and young children are notably lacking, despite their distinct nutritional needs and susceptibility to persuasive techniques. International bodies such as the WHO and UNICEF have identified unhealthy food marketing to infants and young children as a significant public health concern, given its influence on early dietary preferences and long‐term health outcomes (Clark et al. 2020). In Australia, concerns about the composition and marketing of foods for young children have gained increasing attention and are now a priority for government reform (Department of Health and Aged Care Australian Government 2024; Australia and New Zealand Ministerial Forum on Food Regulation 2024). The primary aim of this study was to explore how child‐appealing marketing strategies on commercial foods for infants and young children in Australia have evolved, comparing data from 2015 and 2024. The secondary aim was to assess changes in child‐appealing marketing across different food categories, packaging types, and levels of processing.

## 2 Methods

### 2.1 Data Source

This study used FoodSwitch data from 2015 and 2024. FoodSwitch, developed by The George Institute for Global Health, is a monitoring database that tracks changes in packaged foods, including nutrition, ingredients, and on‐pack claims. Data are collected annually from the four largest Australian grocery retailers: Woolworths, Coles, Aldi, and Independent Grocers of Australia (IGA) (The George Institute for Global Health 2023), covering approximately 90% of the market share for retailers selling packaged groceries in Australia (Jones et al. 2018). Each year, information from approximately 30,000 products is captured, supported by photographs of product packaging (Coyle et al. 2022).

This analysis included products sold in the baby food aisle of supermarkets, but excluded infant and follow‐up formulas, which are regulated separately. Products were excluded if they lacked an on‐pack ingredients list or the photographic set was incomplete in the FoodSwitch database (e.g., not all sides of the product were captured during collection).

### 2.2 Food Categorisation

**Figure 1:**
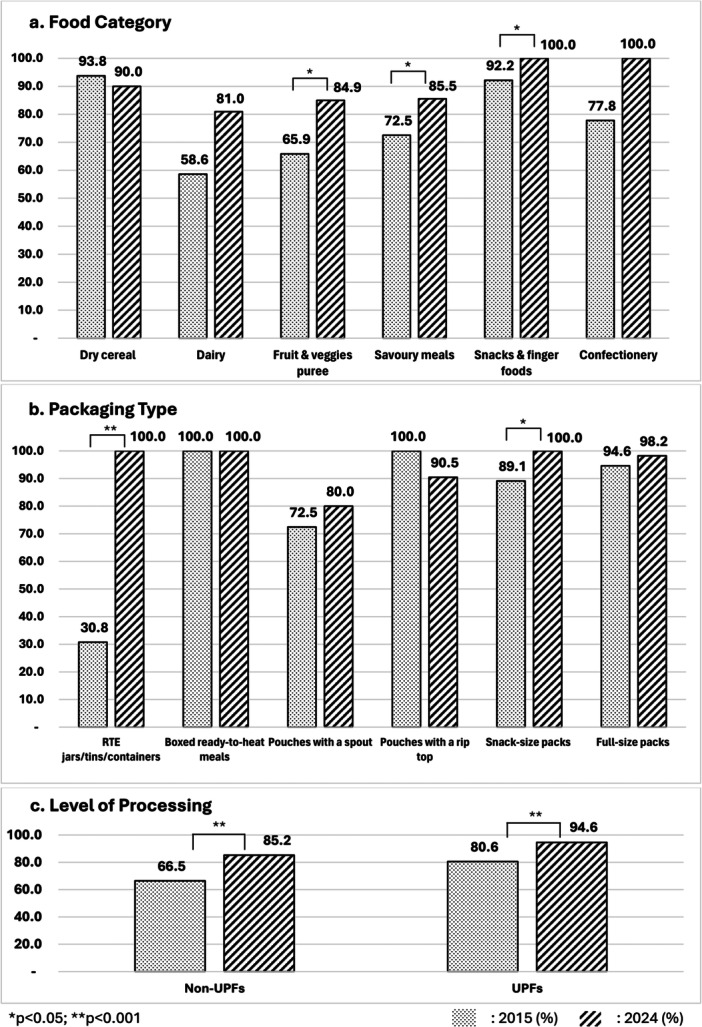
Changes in the presence of child‐appealing marketing techniques across different categories: (a) Food category, (b) Packaging type and (c) Level of processing.

Foods were categorised into seven groups according to the WHO NPPM (World Health Organization Regional Office for Europe 2022). These seven groups were adapted from the original eight NPPM groups, excluding the drinks category because formula products were outside the scope of this study. A visual representation of this categorisation is provided in Supporting Information S1: Figure 1.

### 2.3 Food Packaging Classification

Each food product was categorised into one of six packaging types, adapted from prior research (Dunford et al. 2024). These packaging types included: ready‐to‐eat (RTE) jars/tins/containers, boxed ready‐to‐heat meals (chilled/frozen trays), pouches with a spout, pouches without a spout, snack‐size packs (dry individually wrapped small packs either in multi‐pack form or individually), and full‐size packs (packages with multiple serves provided in packs for dry foods).

### 2.4 Tool to Assess the Use of Child‐Appealing Marketing Techniques

This study adapted a validated coding framework for Child‐Appealing Packaging (CAP), originally developed and validated (Mulligan et al. 2021) and subsequently operationalised for large‐scale packaging analyses (Mulligan et al. 2023), to identify, categorise, and measure the power of child‐appealing marketing techniques. Under the umbrella of child‐appealing marketing, the framework classifies techniques into two categories: core techniques and broad techniques. Core techniques directly target children's interests, for example, using images of cartoon characters or toys on the packaging. Broad marketing techniques do not directly target children but rather appeal to parents and caregivers through a range of techniques, including the use of nutrition and health claims, recipes and convenient packaging [for a full list of child‐appealing techniques and their classifications, see Supporting Information S1: Table 1].

For this study, adaptations were made to the validated CAP framework that applies to children broadly to enable its use specifically for assessing foods for infant and young children where visual features (e.g. bright colours, playful fonts, engaging graphics and characters) are more salient than words for example in populations that cannot yet read. Drawing on Jean Piaget's theory of cognitive development (Piaget and Cook 1977), which highlights young children's focus on visual and imaginative elements, specific codes within both core and broad categories were modified accordingly. Details of these modifications are provided in Supporting Information S1: Table 1.

Each product was assessed against eleven core and nine broad marketing techniques. The analysis focused on three primary outcomes: presence, type, and power of CAP. Presence was defined as the appearance of at least one core marketing technique on the packaging, consistent with prior studies (Mulligan et al. 2021, 2023). Type referred to the specific techniques used; each core and broad technique was evaluated individually using a binary scoring system (1 if present, 0 if absent). Power referred to the overall persuasive strength of the marketing techniques used. It was evaluated by summing the scores of all identified core and broad techniques, yielding a maximum possible score of 20 (11 from core techniques and 9 from broad techniques). A higher cumulative power score indicates greater persuasiveness or intensity of child‐appealing marketing overall.

The scoring technique for the CAP tool followed the staged coding approach, whereby two coders independently assessed and coded 10% of the sample. Inter‐rater agreement was then calculated. If the agreement surpassed a pre‐established threshold (85%), the remainder of the dataset was coded by a single coder. For codes with agreement below the threshold, discrepancies were discussed between the two coders and, when necessary, resolved with input from a third coder. Once consensus was reached, the relevant items were recoded to finalise the dataset (Jones et al. 2023).

### 2.5 Level of Processing

Introduced in 2009, the Nova classification system groups foods and beverages into four categories based on their level of processing (Monteiro et al. 2019): (1) unprocessed or minimally processed (e.g., dried, milled, chilled); (2) processed culinary ingredients (e.g., oils, butter, sugar, salt used in cooking); (3) processed foods (made by adding salt, oil, or sugar); and (4) ultra‐processed foods (UPFs), which contain industrial ingredients and multiple additives, such as sodas, packaged snacks, and ready meals (Monteiro et al. 2019).

In this study, products were classified using the Nova classification system by identifying markers and ingredients of ultra‐processing. These markers include ingredients not commonly used in home cooking, for example, emulsifiers, colourings, flavourings, varieties of sugars, artificial sweeteners, modified oils, protein isolates, and are not limited to this list. In contrast, vitamins, minerals, live cultures, and acids or acidity regulators that do not alter flavour or texture are not considered markers of ultra‐processing (Monteiro et al. 2019). Products containing at least one ultra‐processing marker were classified as Nova group 4. Classification was conducted at the product level, following methods from previous studies (Barrett et al. 2023). For the analysis, we used a binary indicator to distinguish between UPFs (Nova 4) and non‐UPFs (Nova 1–3).

### 2.6 Statistical Analysis

We assessed the number and proportion of products displaying each core and broad child‐appealing marketing technique in 2015 and 2024. To compare differences between the 2 years, we performed two proportion z‐tests. To address the secondary aim, we examined changes in the presence (%) and median power (with Interquartile Range [IQR]) of child‐appealing marketing overall and stratified by food category, packaging type, and level of processing. Two proportion z‐tests were used to compare the differences in the presence, and a Mann–Whitney U test was used to test the difference in median power scores of marketing techniques. Data management was conducted in Microsoft Excel, and statistical analyses were performed using Stata version 19. All statistical tests were two‐tailed, with a significance level set at *p* < 0.05.

### 2.7 Ethics Approval

Ethics approval was not required for this study, as it involved secondary analysis of an existing dataset owned by The George Institute for Global Health. Approval to access and use the data was obtained from The George Institute for Global Health before commencement of the study.

## 3 Results

A total of 313 products were identified for 2015 and 312 for 2024. After excluding products with incomplete data (2 = 2015, 14 = 2024), the final sample sizes were 311 products in 2015 and 298 in 2024 (Table 1). In 2015, the most prominent product types were fruit and vegetable purees (40.5%), savoury meals (25.7%), and snacks and finger foods (16.4%). In 2024, the same three categories remained the most prominent, with fruit and vegetable purees (31.2%), snacks and finger foods (28.2%), and savoury meals (25.5%). However, fruit and vegetable purees decreased by 9.3 percent, and snacks and finger foods increased by 11.8 percent compared with 2015.

**Table 1:** Sample characteristics.

Characteristic	2015	2024
	*n* (%)	*n* (%)
Total number of products	311	298
Food product category		
Dry cereal	16 (5.1)	10 (3.4)
Dairy	29 (9.3)	21 (7.1)
Fruit & Vegetable Puree	126 (40.5)	93 (31.2)
Savoury meal	80 (25.7)	76 (25.5)
Snacks & finger food	51 (16.4)	84 (28.2)
Confectionery	9 (2.9)	14 (4.7)
Packaging type		
RTE jars/tins/containers^a^	52 (16.7)	9 (3.0)
Boxed ready‐to‐heat meals	16 (5.1)	9 (3.0)
Pouches with a spout	149 (47.9)	135 (45.3)
Pouches with a rip top (wet product)	11 (3.5)	21 (7.1)
Snack‐size packs	46 (14.8)	68 (22.8)
Full‐size packs	37 (11.9)	56 (18.8)

^a^ Abbreviation: RTE, Ready‐to‐eat foods are pre‐prepared or processed foods in jars/tins/containers that can be eaten without further cooking or preparation.

According to package type, the most common products in both years were pouches with a spout, accounting for 47.9% in 2015 and 45.3% in 2024. Over the decade, notable shifts in other packaging types were observed. The least common packaging type in 2015 was pouches with a rip‑top, representing only 3.5% of the sample; however, this format increased to 7.1% in 2024. By contrast, in 2024 the least common packaging types were RTE jars, tins and containers (3.0%) and boxed ready‑to‑heat meals (3.0%), both of which declined in use from 2015 (from 16.7% and 5.1% respectively) (Table 1).

Overall, the presence of core marketing techniques increased significantly from 73.0% in 2015 to 89.9% in 2024 (*p* < 0.001) (Table 2). In contrast, the prevalence of broad marketing techniques was already very high in 2015 (99.7%) and reached 100% in 2024, showing no significant change over time (*p* = 0.327).

**Table 2:** Types of marketing techniques used among infant and young children's food products in 2015, compared to 2024.

Child‐appealing marketing techniques	2015	2024	*p* value^a^
	*n* (%)	*n* (%)	
Core marketing techniques	227 (73.0)	268 (89.9)	< 0.001
Child‐appealing visual/graphic design of the package	188 (60.5)	216 (72.5)	0.002
Unconventional shape of the product, featured on the package	3 (1.0)	17 (5.7)	0.001
Unconventional flavour of the product, featured on the package	0 (0.0)	0 (0.0)	—
Unconventional colour of the product, featured on the package	0 (0.0)	0 (0.0)	—
Games or activities on the package	0 (0.0)	0 (0.0)	—
Presence of branded characters or spokespersons	105 (33.8)	186 (62.4)	< 0.001
Presence of licensed characters	0 (0.0)	5 (1.7)	0.022
Presence of celebrities	0 (0.0)	0 (0.0)	—
Other child‐appealing tie‐ins	0 (0.0)	5 (1.7)	0.022
Coupons, contests, or giveaways, specifically appealing to children	0 (0.0)	0 (0.0)	—
Appeals to fun or cool	150 (48.2)	236 (79.2)	< 0.001
Broad marketing techniques	310 (99.7)	298 (100.0)	0.327
Interesting or unconventional product name	22 (7.1)	31 (10.4)	0.145
Presence of a logo/image not specifically appealing to children	306 (98.4)	282 (94.6)	0.011
Promotion of convenient packaging	93 (29.9)	97 (32.6)	0.481
Appeals to taste or texture	297 (95.5)	286 (96.0)	0.772
Appeals to health or nutrition	309 (99.4)	298 (100.0)	0.166
Appeals to other product benefits	156 (50.2)	185 (62.1)	0.003
Recipes	2 (0.6)	0 (0.0)	0.166
Promotion of websites, social media, or rewards programs	88 (28.3)	20 (6.7)	< 0.001
Coupons, contests, or giveaways, not specifically appealing to children	0 (0.0)	0 (0.0)	—

^a^ *p*‐value calculated using the two‐proportion z‐test.

There were notable increases in the use of many core and broad marketing techniques from 2015 to 2024 (Table 2). The most significant increases in core marketing techniques were in the use of appeals to fun or cool (used on 48.2% of products in 2015 vs. 79.2% in 2024), branded characters or spokespersons (33.8% in 2015 vs. 62.4% in 2024) and child‐appealing visual/graphic design of the package (60.5% in 2015 vs. 72.5% in 2024).

Several techniques were consistently absent from commercial foods for infants and young children in both years. These included unconventional flavours or colours, the use of games, celebrity images on packaging, and promotional elements such as coupons, contests, or giveaways.

Among the broad marketing techniques, appeals to other product benefits showed a significant increase, rising from 50.2% in 2015 to 62.1% in 2024. Although other techniques within this category did not change significantly, they were already highly prevalent in 2015 and remained consistently high throughout the decade. For example, appeals to texture or taste were present in 95.5% and 96.0%, respectively; and appeals to health or nutrition appeared in 99.4% and 100%, respectively. Some techniques experienced a significant decline over the same period, including the promotion of websites, social media, or rewards programs (from 28.3% in 2015 to 6.7% in 2024).

Analysis of changes in the presence of child‐appealing marketing techniques between the 2 years is presented in Figure 1. By food category, statistically significant increases were observed in the proportion of products featuring child‐appealing marketing techniques for fruit and vegetable purees (from 65.9% to 85.0%, *p* = 0.002), snacks and finger foods (from 92.2% to 100.0%, *p* = 0.009), and savoury meals (from 72.5% to 85.5%, *p* = 0.047). Although the proportion of products displaying such marketing increased across all packaging types between 2015 and 2024, only two types showed statistically significant rises: ready‐to‐eat jars/tins/containers (from 30.8% to 100.0%, *p* < 0.001) and snack‐size packs (from 89.1% to 100.0%, *p* = 0.005). By level of processing, significant increases were observed for both non‐UPFs (66.5% to 85.2%, *p* < 0.001) and UPFs (80.6% to 94.6%, *p* < 0.001).

Median marketing power rose overall from 5.0 (IQR 4.0–6.0) to 6.0 (IQR 6.0–7.0) (Table 3). This change was statistically significant (*p* < 0.001), indicating a notable intensification in marketing strategies over time.

**Table 3:** Median marketing power^a^ score of infants' and young children's food products in 2015 and 2024, by food category, packaging type and level of processing.

	Median Power Score (IQR)		
	2015	2024	*p*‐value
Overall	5.0 (4.0–6.0)	6.0 (6.0–7.0)	< 0.001
Food category			
Dry cereal	5.0 (4.0–6.0)	5.0 (4.0–6.0)	1.000
Dairy	4.0 (4.0–5.0)	6.0 (5.0–7.0)	< 0.001
Fruit & veggies puree	5.0 (3.0–6.0)	6.0 (5.0–7.0)	< 0.001
Savoury meals	5.0 (3.5–6.0)	6.5 (5.0–7.0)	< 0.001
Snacks & finger foods	5.0 (4.0–7.0)	7.0 (6.0–8.0)	< 0.001
Confectionery	6.0 (6.0–7.0)	7.0 (6.0–7.0)	0.289
Packaging type			
RTE jars/tins/containers	3.0 (3.0–4.0)	6.0 (6.0–6.0)	< 0.001
Boxed ready‐to‐heat meals	6.0 (5.5–6.0)	7.0 (7.0–7.0)	< 0.001
Pouches with a spout	5.0 (4.0–7.0)	6.0 (5.0–7.0)	< 0.001
Pouches with a rip top (wet product)	7.0 (5.0–7.0)	7.0 (6.0–7.0)	0.867
Snack‐size packs	6.0 (4.0–7.0)	7.0 (6.0–7.0)	< 0.001
Full‐size packs	5.0 (4.0–6.0)	6.0 (6.0–7.5)	< 0.001
Level of processing			
Non‐UPFs	5.0 (4.0–6.0)	6.0 (5.0–7.0)	< 0.001
UPFs	5.0 (3.0–7.0)	7.0 (6.0–7.0)	< 0.001

Abbreviations: RTE, Ready‐to‐eat foods are pre‐prepared or processed foods in jars/tins/containers that can be eaten without further cooking or preparation; Non‐UPFs, Non‐Ultra Processed Foods are foods made without industrial ingredients or additives typical of ultra‐processing; UPFs, Ultra Processed Foods are characterised by industrial ingredients and additives not typical in home cooking.

^a^ The dependent variable (Power) is not normally distributed (*p* < 0.005). As a result, we used the Mann–Whitney U test and reported the median and interquartile range.

Changes in median marketing power between the 2 years are shown in Table 3. Across food categories, all except dry cereals and confectionery exhibited significant increases in median marketing power scores between 2015 and 2024 (*p* < 0.001). The largest increases were observed for snacks and finger foods (from 5.0 [IQR 4.0–7.0] to 7.0 [IQR 6.0–8.0]) and savoury meals (from 5.0 [IQR 3.5–6.0] to 6.5 [IQR 5.0–7.0]). All packaging types showed significant increases in median power scores, except for pouches with a rip‐top. The most substantial change occurred for ready‐to‐eat jars/tins/containers (from 3.0 [IQR 3.0–4.0] to 6.0 [IQR 6.0–6.0]), despite decreasing use of this packaging type over the same period. Across both years, marketing presence remained higher among UPFs compared to non‐UPFs. Both non‐UPFs and UPFs demonstrated significant increases in median power scores, with a greater rise observed for UPFs: non‐UPFs increased from 5.0 [IQR 4.0–6.0] to 6.0 [IQR 5.0–7.0], while UPFs rose from 5.0 [IQR 3.0–7.0] to 7.0 [IQR 6.0–7.0] (*p* < 0.001 for both).

## 4 Discussion

This study demonstrates a substantial intensification of child‐appealing marketing on commercial foods for infants and young children in Australia over the past decade. Between 2015 and 2024, both the prevalence and power of marketing techniques increased markedly across almost all product categories, packaging types and levels of processing. Core marketing presence became significantly more common, contributing to a rise in overall marketing presence from 73% to nearly 90% of products. These increases were observed among both non‐UPFs and UPFs, although UPFs continued to display the higher levels of marketing. Broad marketing techniques were already prevalent and remained high in commercial foods for infants and young children. Together, these findings show that child‐appealing marketing on commercial foods for infants and young children has become increasingly powerful, raising concerns about its potential influence on young children's preferences, dietary patterns and long‐term health trajectories.

Our analysis confirms the growth of child‐appealing marketing from already high levels. A 2023 Australian study similarly reported that 87% of commercial foods for infants and young children featured at least one child‑appealing marketing technique, with an average of two techniques on infant products and three on toddler products (Chung et al. 2023). Importantly, our results highlight that these strategies have remained widespread over the last decade, despite global recommendations to restrict the marketing of unhealthy foods to children and to specifically ban child‐appealing marketing on foods for infants and young children (World Health Organization 2010). The persistent prevalence of child‑appealing marketing on foods for infants and young children indicates limited progress in regulating this space. Additionally, our findings of increasing marketing power raise concerns that these strategies may mislead caregivers about nutritional quality, shape early taste preferences, and contribute to unhealthy dietary patterns and obesity risk (Elliott and Truman 2020; Arraztio‐Cordoba et al. 2022).

Specific marketing techniques that have increased most prominently over time include the use of “fun” or “cool” themes and the inclusion of branded characters or spokespersons. These strategies are known to influence children's product preferences and choices, including those of infants and young children (Kraak and Story 2015; Packer et al. 2022). Young children are drawn to imaginative themes, while also struggling to distinguish fantasy from reality, making them especially receptive to persuasive packaging (Piaget and Cook 1977). In terms of broad techniques aimed at caregivers, we observed a rise in claims that emphasise product benefits such as convenience, affordability and sustainability, which are known to shape consumers' evaluations of products and brands, including brand preferences (Liu et al. 2025). Evidence from prior research also shows that when food products for infants and young children align with caregivers' expectations, such as reputation, value and convenience, caregivers report higher satisfaction and are more likely to repurchase and build brand loyalty (Román and Sánchez‐Siles 2018). While this can make decision‐making easier for caregivers, it may also result in repeatedly buying familiar products that do not meet optimal nutritional standards for young children (Bisschoff and Bester 2018). The combined use of child‐focused visuals and parent‐oriented messaging makes packaging especially persuasive at the point of purchase. This dual targeting raises concerns about the cumulative influence of these techniques, particularly their role in normalising the selection of less nutritious foods and shaping dietary behaviours during early childhood.

Our analysis of marketing power or intensity across food categories, packaging types, and processing levels shows higher exposure in 2024 compared with 2015, suggesting that underlying systemic issues within the food supply chain have persisted over the past decade. This pattern suggests that commercial competition and profit‐driven strategies are contributing to the widespread promotion of unhealthy food products in this sector (Food and Agriculture Organization of the United Nations FAO 2024). These dynamics present significant challenges to public health initiatives that aim to create healthier food environments (Grigsby‐Duffy et al. 2022). Although these marketing practices are effective in driving sales, they are associated with substantial hidden health costs. Diets shaped by the promotion of energy‐dense, nutrient‐poor foods are linked to the growing incidence of non‐communicable diseases such as heart disease, stroke, and diabetes (Food and Agriculture Organization of the United Nations FAO 2024).

A significant finding is the consistently higher marketing intensity observed among UPFs compared with non‐UPFs. This pattern was evident in both years and suggests that the most heavily marketed products in the infant and young children's food category are also those of the poorest nutritional quality, reflecting the rapid global expansion of UPFs. A landscape analysis on market data confirms sustained growth in both developed and developing regions, and UPFs now represent a substantial and increasing share of children's diets (Dunford and Popkin 2023). Despite being characterised by the use of additives and industrial ingredients linked to metabolic, endocrine, and developmental harms (Lane et al. 2024; Soni et al. 2024), they are often positioned as practical or appropriate choices through a strong marketing presence. Such strategies may normalise UPF consumption during critical developmental periods, shaping long‐term food preferences and eating habits. Without stronger regulation, promotional strategies may continue to boost demand for UPFs during children's vital growth periods, influencing their food preferences and dietary habits that could last a lifetime (Dunford and Popkin 2023; Lane et al. 2024; Soni et al. 2024).

The rise in marketing observed in our study highlights that existing regulations do not adequately address marketing elements such as fun characters or many of the claims on packaging (Food Regulation Standing Committee 2025). While nutrition content and health claims must be substantiated under current regulations, many other claims (e.g. on sustainability, or affordability) fall outside regulatory oversight. Importantly, best‑practice recommendations such as the WHO NPPM advise that even regulated claims are inappropriate for use on foods for infants and young children (World Health Organization Regional Office for Europe 2022). Marketing techniques on product packaging are also beyond the scope of voluntary guidelines on marketing to children from the national advertising industry's self‐regulatory body (Australian Association of National A 2025). However, even if packaging were included, there is no evidence that voluntary, industry self‑regulatory approaches have worked to protect children (World Health Organization 2023b). This regulatory gap leaves children and their caregivers exposed to marketing practices that may influence perceptions and choices in ways that are not aligned with national and international dietary recommendations (World Health Organization 2023a; National Health and Medical Research Council NHMRC 2013). Lessons from Chile, where the Food Labelling and Advertising Law bans a wide range of child‐appealing marketing on the packaging products “high‐in” sugar, salt, saturated fat or calories, demonstrate the potential of stricter regulation (Taillie et al. 2019). These restrictions primarily focus on core techniques that directly target children, such as characters, games and other child‐appealing visuals. In addition, the law indirectly constrains broad, caregiver‐targeted techniques through restrictions on certain nutrition and marketing claims and the mandatory use of prominent front‐of‐pack warning labels on “high‐in” foods (Taillie et al. 2019). Evaluations suggest these measures reduced child‐appealing marketing and increased awareness among caregivers, positively influencing purchasing and eating behaviours (Taillie et al. 2020). These findings point to opportunities for Australia to strengthen its regulatory approach.

Strengths of this study include the use of a comprehensive dataset from major food retailers in Australia, collected systematically in both years sampled. To our knowledge, this is the first study to examine changes in commercial foods for infants and young children in Australia over the past decade. It provides valuable insights into evolving marketing practices to inform policymakers and stakeholders working to protect children's health. However, a key limitation is its reliance on cross‐sectional data from only two time points, limiting our ability to draw causal inferences. Given the subjective nature of assessing child‐appealing marketing techniques, we minimised potential bias through double‐coding and a more detailed interpretation of the coding framework. Future research could investigate causal relationships between packaging‐based marketing, dietary behaviours, and health outcomes in early childhood.

## 5 Conclusion

This study shows a significant increase in the prevalence and intensity of marketing on the packaging of commercial foods for infants and young children in Australia between 2015 and 2024. Marketing techniques became more prominent and widespread across product categories, packaging types, and processing levels, with ultra‐processed foods consistently showing the highest intensity. Claims on pack aimed at caregivers also remained highly prevalent, reinforcing the influential role of packaging. There is a growing need to strengthen regulation to address all forms of on‐pack marketing on commercial foods for infants and young children, including both child‐targeted and caregiver‐targeted techniques, particularly on unhealthy foods.

## Author Contributions

Prisca Petty Arfines, Daisy Coyle, Eden Barrett, Jennifer McCann, Elizabeth Dunford and Alexandra Jones designed the study. Prisca Petty Arfines and Alexandra Jones developed the methodology. Prisca Petty Arfines and Eden Barrett performed the formal analysis. Eden Barrett and Jennifer McCann conducted the investigation. Prisca Petty Arfines wrote the original draft. Daisy Coyle, Eden Barrett, Jennifer McCann, Elizabeth Dunford and Alexandra Jones reviewed and edited the manuscript.

## Conflicts of Interest

The authors declare no conflicts of interest.

## Supporting information


**Figure S1:** Food categorisation tree for commercial foods for infants and young children based on the World Health Organization Nutrient and Promotion Profile Model (30).
**Table S1:** Outcome variables of the child‐appealing packaging (CAP) coding tools.
**Table S2:** Original and modified codes for core marketing techniques used in this study (31).
**Table S3:** Original and modified codes for broad marketing techniques used in this study (31).
