# Supplementary material for: A Growing Problem: Increases in Child‐Appealing Marketing on Infant and Young Children's Foods in Australia (2015 vs 2024)
Source: Matern Child Nutr. 2026 Jul 14;22(3):e70226. doi: 10.1111/mcn.70226 (PMC13369571; doi:10.1111/mcn.70226)
Supplement: Supplementary file 1 — Figure S1: Food categorisation tree for commercial foods for infants and young children based on the World Health Organization Nutrient and Promotion Profile Model (30). Table S1: Outcome variables of the child‐appealing packaging (CAP) coding tools. Table S2: Original and modified codes for core marketing techniques used in this study (31). Table S3: Original and modified codes for broad marketing techniques used in this study (31). [file MCN-22-e70226-s001.docx]

**Supplementary Files**

**Supplementary Figure 1.** **Food categorisation tree for commercial foods for infants and young children based on the World Health Organization Nutrient and Promotion Profile Model (30)**

**Supplementary Table 1. Outcome variables of the child-appealing packaging (CAP) coding tools**

| **Outcome Variable** | **Explanation** | **Details and categorisation** |
| --- | --- | --- |
| **Presence of child-appealing marketing** | Assesses whether the product packaging is child-appealing by analysing the display of core marketing techniques. | Binary Variable  1: Yes (child-appealing packaging) if ≥1 core marketing technique displayed.  0: No (not child-appealing): 0 core techniques displayed). |
| **Type of child-appealing marketing** | Determines which specific type(s) of core or broad marketing technique(s) are being shown. | Presence (binary: yes/no) or frequency (count) of individual core or broad marketing techniques displayed within a sample. |
| **Power of child-appealing marketing** | Assesses the strength/power (intensity) of the marketing claims based on the number of unique core and broad marketing techniques shown. | Marketing power score (count variable): Total of all the unique core and broad techniques shown on the package (e.g., 1,2,3, etc.). Maximum value for power score 20, consisting of core marketing: 11, and for broad marketing techniques: 9. |

**Supplementary Table 2. Original and modified codes for core marketing techniques used in this study (31).**

| # | Technique | Original | Modification in interpretation |
| --- | --- | --- | --- |
| 1 | Child-appealing visual/graphical design of the package | The packaging features bright colours, playful themes (e.g. fantasy, adventure, sports), or unbranded cartoon-style characters that appeal to children. | Since young children can’t read, packaging is assessed for features like bright colours, playful graphics, child-like fonts or non-branded cartoon-style characters, excluding realistic images. |
| 2 | Unconventional shape of the product, featured on the package | The packaging features an unconventional product shape that differs from the typical form for that product type | ‘Featured on the packaging’ refers to words and/or images that emphasise the product’s shape. |
| 3 | Unconventional flavour of the product, featured on the package | The packaging features an unconventional or non-discernible flavour not typically associated with the product type. | Descriptive taste words that are not actual flavours, including negative expressions (e.g. scrummy, yummy, weird, crazy), were excluded. |
| 4 | Unconventional colour of the product, featured on the package | The packaging features an unconventional colour that is unusual for the product type. | This technique excludes typical product colours and those matching the main ingredient, such as pink for strawberry, green for spinach. |
| 5 | Games or activities on the package | The packaging features games or activities to engage children. | This includes games or fun activities for children placed on the back or side of the packaging. |
| 6 | Presence of branded characters or spokespersons | The packaging features of company- or brand-owned characters that may appeal to children. | Company- or brand-owned characters are identified by a trademark symbol (™ or ®), consistent use across brand marketing, or packaging that names them as mascots. |
| 7 | Presence of Licensed Characters | The packaging features of characters from TV shows, movies, books, or similar media that are likely to appeal to children. | Licensed characters are identified by phrases like “Licensed by” or “Used under license from,” their origin in TV, movies, or books, promotional use, and appearance across multiple brands. |
| 8 | Presence of celebrities | The packaging features of actors, athletes, musicians, or other public figures are likely to appeal to children. | (no modification) |
| 9 | Other child-appealing tie-ins | The packaging features of child-appealing tie-ins to movies, TV shows, sports, etc., are advertised on the package beyond the characters or celebrities already described. | (no modification) |
| 10 | Coupons, contests, or giveaways, specifically appealing to children | The packaging features of coupons, contests, or giveaways promoted on the packaging for later entry or redemption. | (no modification) |
| 11 | Appeals to fun or cool | The packaging features that appeal to fun, humour, enjoyment, happiness, or coolness are associated with consuming the product. | Given infants’ and young children’s limited reading ability, this code captures visual elements that convey happiness, fun or imaginative play through non-verbal cues like smiling characters, playful scenes or adventurous settings. |
| 12a | Promotion of websites, social media, rewards programs, specifically appealing to children | Usage of packaging that promotes websites, social media, memberships, rewards, or sharing opportunities in ways that are appealing to children. | As infants and young children cannot read, we have removed this code and merged it under broad marketing technique #10. |

^a.^Code dropped due to merging with other code

**Supplementary Table 3. Original and modified codes for broad marketing techniques used in this study (31).**

| # | Technique | Original | Modification in interpretation |
| --- | --- | --- | --- |
| 1^a^ | Interesting font or lettering | Packaging features a product name or description in a colourful, creative or playful font that, while not enough alone to be deemed child-appealing, may contribute to the overall marketing power; stronger cases fall under core technique #1. | We acknowledge overlap with Code 1 of the core marketing techniques, where lettering is a key aspect of visual evaluation, and have therefore removed this code in line with expert recommendations. |
| 2 | Interesting or unconventional product name | Packaging features an unconventional product name, such as rhyming, alliteration or unusual spelling, that may appeal to children and enhance marketing impact. | We only assessed the product name, not including the brand name. |
| 3 | Presence of a logo/image not specifically appealing to children | Packaging features a product or brand logo, or images such as cartoons, families, or children that are not specifically child-appropriate. | All images not covered by core marketing techniques and not sufficiently child-appealing fall under this code, including realistic illustrations, brand logos, and photos of food, fruits, vegetables, animals or children. |
| 4 | Promotion of convenient packaging | Packaging features specific promotion of convenience or ease of use, excluding single-serve formats without explicit convenience claims. | This code covers packaging-related claims highlighting ease of use, handling or preparation, including variety packs, portable formats, resealable features or statements suggesting convenience for children or caregivers. |
| 5 | Appeals to taste or texture | Packaging features appeal to the product’s flavour, taste or texture in a way that is not specifically child appealing. | This code includes words describing taste, such as scrummy, yummy, or gourmet, including negative taste descriptions like weird or wacky. |
| 6 | Appeals to health or nutrition | Packaging features health or nutrition claims, symbols, or imagery suggesting benefits like growth, strength, or physical activity, including organic, natural, or “healthy food” associations. | This code also includes packaging-related health claims such as “BPA free,” “no added colours or preservatives,” “GMO free,” and allergen-free claims like “nut-free” or “lactose-free.” |
| 7 | Appeals to other product benefits | Packaging features appeal to non-health, non-taste, and non-fun benefits such as value, convenience, sustainability, philanthropy or religious certifications, excluding minor statements about recyclability. | This code excludes mandatory country-of-origin labelling but includes award logos, affordability, sustainability, philanthropy, religious certifications, and ingredient premiumisation claims that emphasise quality or exclusivity over health. |
| 8 | Recipes | Packaging features recipes using the product, excluding standard cooking instructions for preparation-required items. | Standard cooking instructions or serving tips, such as recipes on baby cereals, are excluded from this code. |
| 9 | Promotion of websites, social media, or rewards programs | Packaging features promotion of websites, social media, memberships, rewards or sharing opportunities that are not specifically child-appealing. | Given infants' and young children’s limited reading ability, this code captures text with marketing intent linking to websites or social media, excluding cases where such links are listed solely as contact information. |
| 10 | Coupons, contests, or giveaways, not specifically appealing to children. | Packaging features coupons, contests or giveaways for later redemption that are not specifically child appealing. | No modification |

^a.^Code dropped due to merging with other code
